# Trends of maxillofacial trauma: An update from the prospective register of a multicenter study in emergency services of Chile

**DOI:** 10.4317/medoral.22985

**Published:** 2019-08-19

**Authors:** Fabiola Werlinger, Marcelo Villalón, Valentina Duarte, Raúl Acevedo, Rodrigo Aguilera, Diego Alcocer, Mario Arriola, Oscar Badillo, René Briones, Cristian Condal, Marcela Del Río, Jaime Henríquez, Roberto García, Mauricio Herrera, Joaquín Jaramillo, Felipe Merchan, Marco Nasi, Roberto Osbén, Alejandro Rivera, Santiago Riviello, Patricio Rojas, Constanza Vidal, Gastón Rodríguez, Sebastián Schild, Esteban Arroyo, María-José Alvarado, Pilar Sepúlveda, Juan Cortés

**Affiliations:** 1 Center of Epidemiology and Oral Disease Surveillance (CEVEO), Faculty of Dentistry, University of Chile. Santiago, Chile; 2 Faculty of Medicine, Department of Medical Thecnology, University of Chile. Santiago, Chile; 3 PhD’s student in Methodology of Biomedic Research and Public Health, Universitat Autònoma de Barcelona. Barcelona, Spain; 4 Faculty of Medicine, School of Public Health , University of Chile. Santiago, Chile; 5 Pontificia Universidad Católica de Valparaíso. Valparaíso, Chile; 6 Hospital Carlos Van Buren. Valparaíso, Chile; 7 Hospital Dr. Sótero del Río. Santiago, Chile; 8 Hospital Dr. Gustavo Fricke. Viña del Mar, Chile; 9 Faculty of Dentistry, University of Chile. Santiago, Chile

## Abstract

**Background:**

Determine the behavior of the maxillofacial trauma of adults treated in 3 tertiary care centers in the central zone of Chile.

**Material and Methods:**

Descriptive, cross-sectional, multicenter study, based on the prospective records of maxillofacial trauma cases attended between May 2016 and April 2017 by dental and maxillofacial clinical teams of Adult Emergency Units of hospitals Dr. Sótero del Río (metropolitan region), Carlos Van Buren and Dr. Gustavo Fricke (region V). Age, sex, date of occurrence, type of trauma according to ICD-10, etiology, legal medical prognosis and associated injuries were recorded, stratifying by sex and age. Chi square and unpaired Wilcoxon tests were used to compare by groups.

**Results:**

2.485 cases and 3.285 injuries were investigated. The male: female ratio was 1.7: 1 with age under 30 predominant, followed by older adults. Variability was observed in the yearly, weekly and daily presentation. The highest frequencies were in January and September, weekends and at night. The main etiologies were violence (42.3%), falls (13.1%) and road traffic crashes (12.9%) with differences by age and sex (*p*<0.05). 31,9% of the injuries occurred in hard tissue, being fractures in nasal bones predominant (S02.2).

**Conclusions:**

The profile of the maxillofacial trauma in Chile seems to be mixed by age, affecting young people and the elderly. The male sex predominates; the main cause, which varies by age group, is violence. Their surveillance is possible from hospital emergency records.

** Key words:**Maxillofacial trauma, emergency department, multicenter study.

## Introduction

In several countries of the world, trauma events maintain a shared relevance with chronic non-communicable diseases (1). In this profile of morbidity and mortality, trauma appears as a kind of “neglectec epidemic”, with a high demand for medical care and rehabilitation services that requires standardized protocols for its diagnosis and registration, in order to establish its magnitude and monitoring more accurately (2). Despite these requirements, current trends indicate that health expenditures devoted to research are scarce, with the consequent lack of information for adequate intervention (2,3).

The maxillofacial trauma is a cause for consultation corresponding to more than 4% of the emergency cases (4) and up to a quarter of the polytraumatized cases (5). The challenges for its adequate control are complex since it requires a more comprehensive intervention that involves different sectors, not only health (2). Currently, its incidence has increased, presenting a variable and dynamic behavior that would go hand in hand with the evolution of the different conditions of the population (2,6,7). This would lead to significant differences, even within the same region (8,9) with profiles and mechanisms differentiated by sex and age (2,9,10,11). In developing countries in the Middle East and Africa, for example, profiles are strongly dominated by men (over 75%), between 20 and 30 years and the main etiology is road traffic crashes (2,7,12-14). In countries of Europe and North America, the proportion between men and women tends to narrow, with older ages of presentation (between 30 and 40 years old) and the main etiologies are violence (2,4,15) or falls (4,8,9,16). This difference is attributed in part to the aging of the population experienced by higher income countries (15,16) and to the successful implementation of road legislation measures in response to the main cause identified to control its incidence (2,15,17). The context described has motivated the WHO to promote, since 1996, the increase in funding for research in the area of traumatology, pointing it out in its Priority Program on Disease Control in Developing Countries (DCPDC), as an “Optimal investment” considering it a public health problem due to its magnitude and severity (2,3).

In Chile, until now, there are studies with a clinical predominance aimed essentially to diagnose fractures. In that regard, this proposal seeks to determine the behavior of the maxillofacial trauma in adults from a population perspective, based on the emergency records of three tertiary hospitals.

## Material and Methods

Observational, descriptive and multicenter study, which included the universe of incidents of maxillofacial trauma in adults registered between May 1, 2016 and April 30, 2017, attended by the clinical teams of the Emergency Departments of three tertiary hospitals: Dr. Sótero del Río Hospital (Santiago), Carlos Van Büren Hospital (Valparaíso) and Dr. Gustavo Fricke Hospital (Viña del Mar). The centers were selected for convenience based on: 1) the availability of maxillofacial surgeons for emergency care, 2) the availability of emergency care in situ 24 hours a day and 3) the total population assigned to each hospital (around 3.000.000 inhabitants).

We included the records of all patients older than 18 who consulted for maxillofacial trauma spontaneously or by referral, excluding those with dentoalveolar trauma as a single lesion, those resulting from sequelae, complications of treatment or with missing information in the variables of interest of the study. The data were collected with the inspection of the registration forms of each center, including manual and electronic versions. Due to this variability, a pilot review was carried out, in which each modality was compared by selecting the fields or homologous variables required in the study. They considered sex, age, time, day and month of occurrence, etiology, diagnosis of trauma, legal medical prognosis of the lesion and presence of associated injuries. For the selection of cases, the research team established a standardized criterion (18,19) to extract the matching diagnoses in the fields, items or columns of the sections of “diagnosis”, “observations” and “indications”, or their homologous among the centers. For the paper records, each piece of information was extracted anonymously, directly from the collection point. As for the electronic records, the respective informatics units delivered all the cases with maxillofacial surgery. In this stage, the identification of each case was made with an exhaustive review of the variables included in the diagnostic dimension, excluding those related to the identification of the patient. This process was supported by the automated search for keywords such as: “trauma”, “injury”, “blow”, “fall”, “aggression”, “attack”, “accident”, “facial”, among others. The process was executed by the project’s monitoring team, consisting of the responsible researcher, a specialist in public health and two maxillofacial surgeons. Subsequently, the diagnosis was harmonized with the International Classification of Diseases (ICD), version 10. This involved the selection of the diagnosis, identifying the various nomenclatures and terminologies used by the clinical teams as literal terms, synonyms, technical jargon, neologisms and semantic equivalences of health care language. The terminological concordance with the ICD 10 code was made considering a univocal relation of each term extracted with a concept of international classification. Additionally, the variables corresponding to the temporal occurrence of the trauma were recorded as “diurnal” when the time of occurrence was between 08:00 a.m. and 19:59 p.m. and “nocturnal” if it happened between 20:00 p.m. and 07:59 a.m.; and the classification of the etiology was made using the categories suggested by the WHO in the chapter on Injuries and Violence (2), including in “others” the traumas suffered in the work environment, sports or animal attacks.

Once the final database was configured, a uni and bivariate descriptive analysis was performed, stratifying by sex and age. In this phase, the Chi square and unpaired Wilcoxon tests were applied, with a level of significance of 5% in the Stata 14.0® software. To evaluate the normal distribution of age, the Shapiro-Wilk test was considered. The project was reviewed and approved by the Scientific Ethics Committee of the Faculty of Dentistry of the University of Chile.

## Results

**Figure 1:**
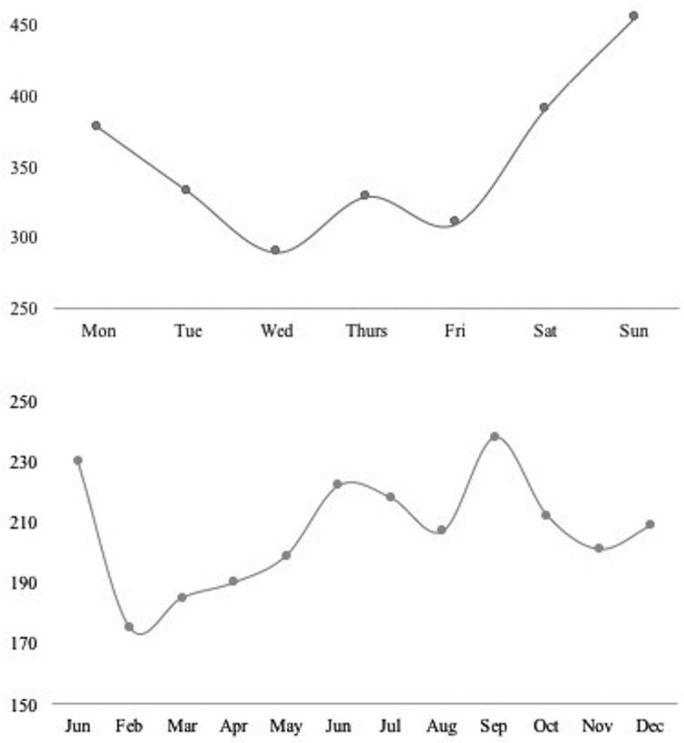
Weekly and annual occurrence of maxillofacial trauma cases.

**Figure 2:**
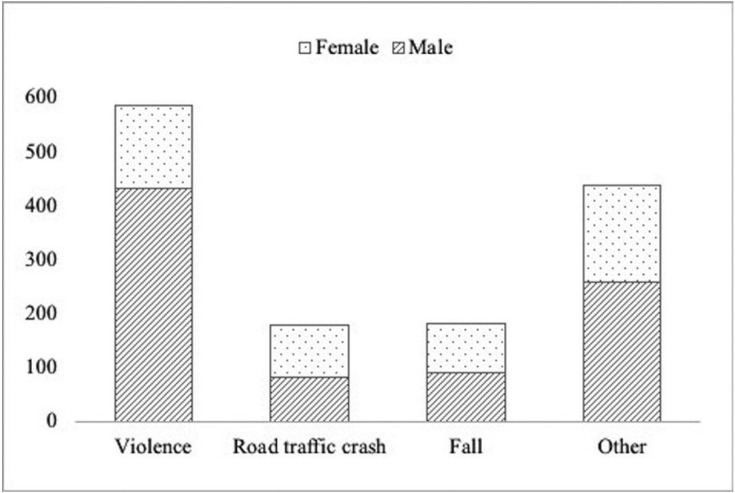
Etiology of maxillofacial trauma cases according to sex.

**Figure 3:**
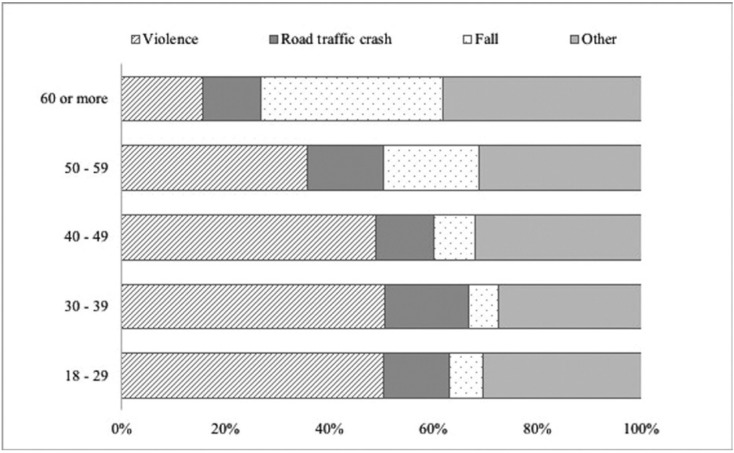
Etiology of maxillofacial trauma cases according to age group.

During the study period, a total of 2.485 consultants were registered for maxillofacial trauma with 3.285 consigned injuries. The male: female ratio was 1,7:1 with a median age of 34 (RIC: 25-51) for men and 44 (RIC: 27-67) for women (*p*=0,001). When disaggregated by age group, the highest frequency was observed in the group of 18 to 29 years (37,1%), followed by the group greater than 60 (20,6%), 30 to 39 (16,6%), 40 to 49 (13,5%), 50 to 59 (12,2%). Other associated lesions were present in 13,0% of the cases where the legal medical prognosis established by the clinical professional was severe in 15,9%, moderate in 18,9% and mild in 65,1% of the cases ( Table 1). According to the schedule and date of consultation, the behavior of the daily, weekly and yearly occurrence was established. Regarding this, 71,3% (n=1.770) of the attentions were performed at night. The detail by week and year is presented in figure 1. During the study, the etiology of 66,7% of the registered cases could be determined (n=1.385). In this group, the most frequently identified cause was violence (42,3%), followed by falls (13,1%) and road traffic crashes (12,9%). The category “other” included 37,1%. It was possible to verify significant differences in the composition of the etiology by sex (*p* value=0,001) and age distribution (*p* value=0,001). The detail is given in figures 2 and 3.

**Table 1:**
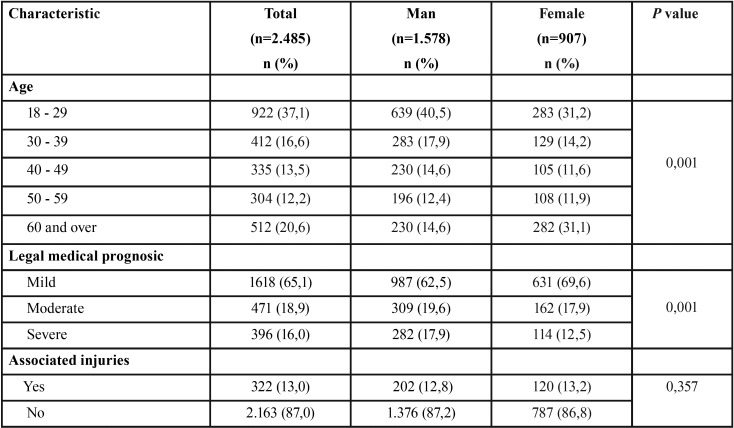
Characteristics of the study population.

Finally, it was established that 68,1% (n = 2.206) of the lesions corresponded to soft tissue and 31,9% (n=1.079) to hard tissue injuries ( Table 2). In the first case, the most frequently diagnosed specified lesion corresponded to “contusion in the eyelid and periocular area” (14,2%), followed by “open wound of lip and oral cavity” (11,4%) and “superficial injury of nose” (11,2%). In the case of hard tissue injuries, the most frequent was the “fracture of nasal bones” (43,9%), followed by “fracture of tooth” (16,1%) and “fractures of malar and maxillary bones” (14,5%).

**Table 2:**
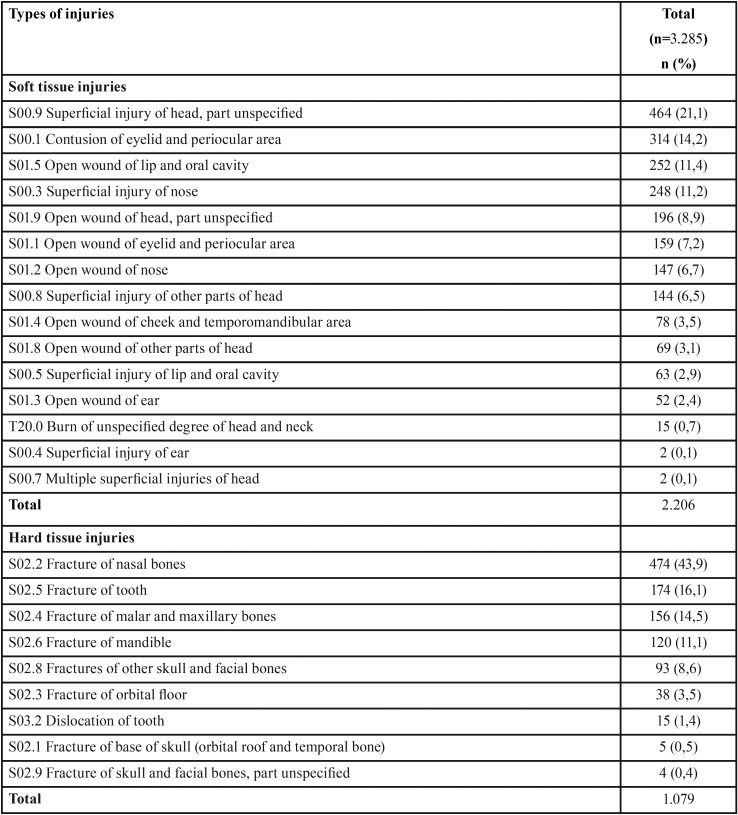
Diagnosis of maxillofacial trauma according ICD-10.

## Discussion

Ours is the first multicenter study in Chile, from a public health perspective, to address the maxillofacial trauma registry in three tertiary hospitals in the most populated regions of the country. In this exploration, it was possible to confirm that a profile composed mainly of young males, the main cause is violence and fracture occurs in 1 out of 3 cases. In this regard, and despite the fact that we only include the adult population (over 18 years of age), our results coincide with that reported by other studies conducted in India, Ireland and Brazil (7,8,20), which position the age group between 20 and 30 years as the most affected by this type of trauma. This has been widely reported in the scientific literature that directly associates the lifestyles of the youngest with risk behaviors that increase the probability of suffering intentional or unintentional injuries (2,3). However, it is also important to note that in our case, one out of every 5 injured is an older adult. This is consistent with the process of population aging experienced in Chile, where those over 60 years of age have shown sustained growth, reaching 10% of the national population (21). These considerations have been collected by countries that, in transitions as advanced as ours, focus their efforts on analyzing this risk group, establishing their main etiologies (8,16) and implementing multifactorial strategies for their approach, such as balance training or correction of visual deficiencies (16). The foregoing considers differential measures for a mixed age profile. It is also possible to observe that the traditional predominance of men over women (2) is more discreet than that reported in other populations (7,12-14) even in our region (20). Clearly, the progressive increase of female participation in activities of the social and work environment explains their greater presence in these events, together with the socioeconomic development that the country is experiencing (22). However, this relationship varies when disaggregated by the origin of the trauma. When the maxillofacial trauma is caused by violence, men are predominant, while in falls and road traffic crashes, there is an equal amount of cases in men and women, which is based on the above and has also been reported by international experience (6,23). In the same way, the composition of the etiology by age group is differential. Those over 60 suffer from maxillofacial trauma mainly due to falls, while in the rest (under 59) it is mainly due to violence. Road traffic crashes are distributed homogeneously among the age groups. The displacement of the latter as the main cause of this type of trauma would be the result of the implementation of various measures in road legislation, as described in the European experience (2,15) which would determine that other etiologies such as violence or falls become the most predominant. In addition, the “uniformity” observed in the casuistry of road traffic crashes would suggest the need to formulate new strategies for its control (2).

When reviewing the time of occurrence, it is possible to observe that, as in other populations, the trauma occurs mostly at night (8,9), privileging weekends (8,9,17) and summer season (9,24). In our case, we also see an increase in the months of January and September, with the lowest frequency in the month of February, all dates included within the spring and summer holiday periods in our country. The fact that February is the month of lowest incidence throughout the year can be explained by the holiday period in the central zone, which is traditionally associated with a temporary migration to other parts of the territory. However, the particular increases of January and September could involve other more complex components as they correspond to dates of national and international celebration (national holidays and new year). These characteristics have been described as a trigger for acts of violence (intentional injuries) (3,8,24) and road traffic crashes (unintentional) (11) where mediators such as alcohol and drugs play an enhancing role (3,8,17,24,25). Unfortunately, the detail of this information, as stated in other studies of a similar nature, is not well documented in the emergency registers (24), a fact that was repeated in our study. The same hypothesis arises when analyzing the distribution of trauma cases throughout the week. There is a concentration of cases between Fridays and Sundays (8,9,10), which can be explained by a greater exposure to injuries, as people practise recreational activities away from home on these days, with a higher probability of consuming harmful substances. (3,8,19,25). It is important to note that, Thursdays are also included in this pattern of behavior.

On the other hand, the fact that most of the traumas investigated in our study correspond to minor soft-tissue injuries, coincides with other regional reports where acts of violence are also the predominant cause (11,17,20) and fractures are associated with major events such as road traffic crashes (7,10,11,17,20,26). Additionally, the presence of cases of maxillofacial trauma with other associated injuries would be an indicator of severity (7,17), present in 1 out of 10 cases in our study. Regarding the profile of the lesions, there is a varied description in the literature, by sex (7,10,17) etiology (6,15,17), age group (10,13), diagnostic form (12) and type of registry (4). In our study, the use of a worldwide nomenclature in the medical field permitted to establish that most of the fractures occurred in the middle third of the face, specifically in the bones of the nose. These results are similar to experiences reported in Turkey (6), Iran (7) and Brazil (20), where violence and road traffic crashes predominate as the main etiologies. At the same time, it is interesting to observe that in the case of soft-tissue injuries, the lesions without a specified anatomical area reached almost a third of the diagnoses, a fact possibly supported by a greater assessment in the registry of hard tissue injuries. This, added to the lack of information regarding the etiology of the events, would indicate the need to reinforce the clinical registry processes by constituting a source of relevant information for the planning of hospital resources (treatment, stay and human resources) in the management of this type of injuries (17).

The aim of this research was to contribute, in a first phase, to the study of maxillofacial trauma from a comprehensive perspective, establishing a situational diagnosis for clinical decision making and public health. For this reason, its design contemplated two regions that together represent 52,3% of the national population (21), in order to achieve greater representativeness. This means that our findings have implications at different levels, raising questions about the strategic planning needed to cover emergency services during the dates and times associated with a higher incidence (24), as well as the proposal of intersectoral policies that reduce road risks, the use of harmful substances and access to firearms, as well as the development of more complex interventions in education aimed at developing programs that foster secure relationships or social skills (2,3). Although the disparity in the forms of registration constitutes a weakness, both in the accurate diagnosis and in the monitoring of this type of events, it guides us towards the development and implementation of new standardized forms of registration in a joint effort between clinical and computer equipment for obtaining complete and consistent evidence. Considering that the maxillofacial trauma corresponds to an acute event, the attention in emergency units not only ensures its rapid intervention but also makes it an ideal place for the registration, due to the temporal and geographical proximity to the event, allowing its continuous monitoring and updating. Thus, our results are sided with the need to permanently update the reports regarding the behavior of the population as one of the guidelines of the reference hospitals.

## References

[1] (2018). The top 10 causes of death. World Health Organization.

[2] (2010). The top 10 causes of death. Traumatismos y violencia. Datos.

[3] Jamison DT, Nugent R, Gelband H, Horton S, Jha P, Laxminarayan R (2018). Prioridades para el control de enfermedades: Compendio de la 3ª edición. Banco Mundial.

[4] Hutchison IL, Magennis P, Shepherd JP, Brown AE (1998). The BAOMS United Kingdom survey of facial injuries part 1: aetiology and the association with alcohol consumption. Br J Oral Maxillofac Surg.

[5] Esmer E, Delank KS, Siekmann H, Schulz M, Derst P (2016). Gesichtsverletzungen bei Polytrauma − Mit welchen Verletzungen ist zu rechnen?. Notfall + Rettungsmedizin.

[6] Arslan E, Solakoglu A, Komut E, Kavalci C, Yilmaz F, Karakilic E (2014). Assessment of maxillofacial trauma in emergency department. World J Emerg Surg.

[7] Rezaei M, Jamshidi S, Jalilian T, Falahi N (2017). Epidemiology of maxillofacial trauma in a university hospital of Kermanshah, Iran. J Oral Maxillofac Surg Med Pathol.

[8] Walker T, Byrne S, Donnellan J, McArdle N, Kerin M, McCann P (2012). West of Ireland facial injury study. Part 1. Br J Oral Maxillofac Surg.

[9] Al-Dajani M, Quiñonez C, Macpherson A, Clokie C, Azarpazhooh A (2015). Epidemiology of Maxillofacial Injuries in Ontario, Canada. J Oral Maxillofac Surg.

[10] De Macedo I, Santos L, Ferreira A, Almeida T, Nóbrega L, D'Avila S (2017). Multiple correspondence analysis as a strategy to explore the association between categories of qualitative variables related to oral- maxillofacial trauma and violent crimes at the community level. Int J Oral Maxillofac Surg.

[11] D'Avila S, Nóbrega K, De Macedo I, Marques L, Meira P, Ferreira E (2016). Facial trauma among victims of terrestrial transport accidents. Braz J Otorhinolaryngol.

[12] Adenike A, Olufemi A, Olukunle T, Olubayo A (2015). Updates on the Epidemiology and Pattern of Traumatic Maxillofacial Injuries in a Nigerian University Teaching Hospital: A 12-Month Prospective Cohort In-Hospital Outcome Study. Craniomaxillofac Trauma Reconstruction.

[13] Singaram M, Vijayabala S, Kumar R (2016). Prevalence, pattern, etiology, and management of maxillofacial trauma in a developing country: a retrospective study. J Korean Assoc Oral Maxillofac Surg.

[14] Weihsin H, Thadani S, Agrawal M, Tailor S, Sood R, Langalia A (2014). Causes and incidence of maxillofacial injuries in India: 12-year retrospective study of 4437 patients in a tertiary hospital in Gujarat. Br J Oral Maxillofac Surg.

[15] Boffano P, Roccia F, Zavattero E, Dediol E, Uglesi V, Kovacic Z (2015). European Maxillofacial Trauma (EURMAT) project: A multicentre and prospective study. J Craniomaxillofac Surg.

[16] Kannus P, Niemi1 S, Parkkari J, Harri Sievänen H (2016). Rising incidence of fall-induced maxillofacial injuries among older adults. Aging Clin Exp Res.

[17] Roccia F, Savoini M, Ramieri G, Zavattero E (2016). An analysis of 711 victims of interpersonal violence to the face, Turin, Italy. J Craniomaxillofac Surg.

[18] (2018). Committee on Trauma of the American College of Surgeons. The Committee on Trauma.

[19] Carvalho T, Cancian L, Marques C, Piatto V, Maniglia J, Molina F (2010). Six years of facial trauma care: an epidemiological analysis of 355 cases. Braz J Otorhinolaryngol.

[20] Domingues L, Da Silveira A, Giacomelli G, Guerra R, Henrique R, Manzolli F (2018). Epidemiology and Risk Factors of Maxillofacial Injuries in Brazil, a 5-year Retrospective Study. J Maxillofac Oral Surg.

[21] (2017). Compendio estadístico. Instituto Nacional de Estadísticas.

[22] (2015). Mujeres en Chile y Mercado del Trabajo. Instituto Nacional de Estadísticas Chile.

[23] Da Rosa A, Granke G, Faot F, Rezende L, Manzolli F, Torriani M (2017). Etiology, diagnosis, and demographic analysis of maxillofacial trauma in elderly persons: A 10-year investigation. J Craniomaxillofac Surg.

[24] Islam S, Uwadiae N, Hayter J (2016). Assault-related facial injuries during the season of goodwill. Oral and Maxillofacial Surgery.

[25] Goulart D, Durante L, De Moraes M, Asprino L (2015). Characteristics of maxillofacial Trauma among alcohol and drug users. J Craniofac Surg.

[26] Marques L, De Macedo I, Nóbrega K, Lira J, Targino A, D'Avila S (2017). Pattern of oral-maxillofacial trauma from violence against women and its associated factors. Dent Traumatol.

